# microRNA-93 promotes cell proliferation via targeting of PTEN in Osteosarcoma cells

**DOI:** 10.1186/s13046-015-0192-z

**Published:** 2015-08-05

**Authors:** Masanori Kawano, Kazuhiro Tanaka, Ichiro Itonaga, Shinichi Ikeda, Tatsuya Iwasaki, Hiroshi Tsumura

**Affiliations:** Department of Orthopaedic Surgery, Faculty of Medicine, Oita University, Oita, 879-5593 Japan

## Abstract

**Background:**

Aberrant microRNA (miRNA) expression plays an essential role in osteosarcoma (OS) pathogenesis. Recent studies have shown that dysregulation of miRNA expression is associated with increased tumorigenesis and poor prognosis in several types of cancers, including OS. The aim of this study was to investigate the relevant microRNAs involved in the development of OS.

**Methods:**

To explore possible oncogenic factors in OS, we used a microarray-based approach to profile changes in the expression of miRNAs and their target mRNAs in five OS cell lines and human mesenchymal stem cells (hMSCs). An miRNA, miR-93, was significantly up-regulated, whereas phosphatase and tensin homologue (PTEN) expression was significantly down-regulated in all tested OS cells, when compared with hMSCs.

**Results:**

When anti-miR-93 was transfected into OS cell lines, PTEN expression was greatly increased, suggesting that PTEN might be a target of miR-93 in ES cells. The expression of phosphorylated Akt protein, which is known to be inversely correlated with that of PTEN, was significantly down-regulated in anti-miR-93-transfected cells. Furthermore, transfection of anti-miR-93 inhibited the proliferation and cell cycle progression of ES cells. In addition, the down-regulation of miR-93 in these cells significantly suppressed tumor growth in vivo.

**Conclusion:**

Ectopic expression of miR-93 decreased PTEN protein levels. Furthermore, miR-93 increased proliferation and decreased apoptosis in OS cells, whereas its silencing in these cells inhibited such carcinogenic processes. Taking these observations together, miR-93 can be seen to play a critical role in carcinogenesis through suppression of PTEN, and may serve as a therapeutic target for the treatment of OS.

## Introduction

Osteosarcoma (OS) is the most common primary malignant bone tumor in children and adolescents. Traditional therapeutic approaches include local control of the primary lesion by surgery and/or chemotherapy, and treatment of disseminated disease with multiagent cytotoxic chemotherapy. However, over the last three decades, there have been no noticeable improvements in patient survival, especially for the subgroup having shown metastasis at diagnosis [1, 2].

MicroRNAs (miRNAs) have been shown to be important post-transcriptional regulators of gene expression in both cancer cells and normal cells. These noncoding small RNAs bind to specific cognate sequences in the 3′-untranslated region (3′-UTR) of target transcripts, usually resulting in translational repression and gene silencing [3, 4].

MiR-93 expression has been implicated in various cancer types, implying an oncogenic role [5–7]. In breast cancer, its overexpression has been correlated with proliferation and tumor progression [8]. However, the role of miR-93 in the proliferation of OS cells remains unclear.

Studies of phosphatase and tensin homologue (PTEN) expression in a variety of malignancies including breast, gastric, esophageal, and uterine cancers have demonstrated that reduced PTEN levels are associated with poor prognoses [9–13].

In the present study, we analyzed genome-wide expression arrays of both miRNAs and mRNAs in five human OS cell lines and human mesenchymal stem cells (hMSCs). Our results indicated that the expression of miR-93 was elevated while that of PTEN was repressed in all five OS cell lines, in comparison to hMSCs. Based on this inverse correlation, we hypothesized that the effect of PTEN in OS cells may be directly or indirectly mediated, at least in part, via miR-93. The purpose of our study was to assess whether the expression of PTEN is repressed by miR-93 and to establish whether this pathway could play a role in tumorigenesis in OS cells.

## Material & methods

### Cell lines

The human OS cell lines—HOS, SaOS, and MG-63—were obtained from RIKEN Cell Bank (Tsukuba, Japan), and NY and Hu09 were obtained from JCRB Cell Bank (Osaka, Japan). hMSCs were purchased from TaKaRa Biotechnology (Otsu, Japan). The genotype and phenotype of each cell line was authenticated by the respective source company. HOS cells were grown in minimal essential medium (MEM) supplemented with 10 % fetal bovine serum (FBS; Invitrogen, NY) and 0.1 mmol/L nonessential amino acids (NEAA). SaOS, MG-63, and NY cells were cultured in a high-glucose medium, Dulbecco’s modified eagle medium (DMEM) (Invitrogen, NY) supplemented with 10 % FBS and 1 % penicillin and streptomycin. The Hu cells were cultured in Roswell Park Memorial Institute medium (RPMI) 1640 (Invitrogen) supplemented with 10 % FBS. hMSCs were cultured with the Chemically Defined Mesenchymal Stem Cell Basal Medium (MSCBM-CD) with MSCGM-CD SingleQuats (TaKaRa Biotechnology). The cells were maintained at 37 °C under 5 % CO2, and passaged every 2–3 days.

### Ethics statement

The animal experimental protocol was approved by the Ethics Review Committee for Animal Experimentation of Oita University, and all mice used in this study were anesthetized with ketamine/xylazine or isoflurane/oxygen for experiments and euthanized with cervical dislocation under anesthesia. All efforts were made to minimize suffering.

### Mice

BALB/c nu/nu mice, (n = 28, 6 week old,), were acquired from the Kyodo Laboratory (Tosu, Japan). After quarantine, all mice were kept in a pathogen free environment on a standard 12 hr-day/12 hr-night cycle and were fed a standard sterilized pellet diet and water ad libitum. All mice were continuously monitored during daytime from Monday to Friday, and twice daily during daytime on Saturdays, Sundays, and holidays for signs of poor health.

### RNA isolation

mRNAs were prepared from the triplicated cell cultures using RNeasy kit (Qiagen, Valencia CA) according to the manufacturer’s instruction. The RNA quality was ensured, before labeling, using RNA 6000 Nano kit and Bioanalyzer 2100 (Agilent, Santa Clara, CA). miRNAs were prepared from triplicate cell cultures using the miRNeasy Mini kit (Qiagen) according to the manufacturer’s instruction.

### Genome-wide miRNA expression microarray

GeneChip miRNA 3.0 array (Affymetrix, Santa Clara, CA) was used for miRNA expression profiling in five OS cell lines and hMSCs. One μg of small RNA including miRNA from each sample was labeled with biotin using the FlashTag Biotin HSR Kit (Genisphere, Hatfield, PA). Array hybridization, washing and scanning of the slides were carried out according to the manufacture’s recommendations. The data were extracted from the images, quantile-normalized, summarized (median polish), and log2-transformed with miRNA QC software (Affymetrix). GeneSpring GX 11.0 (Agilent) was used to analyze the array results. Analysis of variance was used to determine those probe sets significantly different between the two groups. The gene list was filtered with a fold-change cutoff of 2 resulting in the output of list with genes that have significant differential expression at 2-fold or more differences. Pathway analysis was performed using KEGG PATHWAY Database (http://www.genome.jp/kegg/pathway.html).

### Analysis of mRNA expression by cDNA arrays

GeneChip Genome HG U133 Plus 2.0 Array (Affymetrix) was used for mRNA expression profiling in five OS cell lines and hMSC. Biotinylated cRNA was synthesized from total RNA using the 3′ IVT Express Kit (Affymetrix) according to the manufacturer’s protocols. In brief, double stranded cDNA was generated by reverse transcription from 1 ng of total RNA an oligo (dT) primer bearing a T7 promoter. The double-strand cDNA was used as a template for in vitro transcription to generate biotin-labeled cRNA. After fragmentation, 12.5 μg of cRNA were hybridized to GeneChip array for 16 hr. The arrays were washed and stained using GeneChip Fluidics Station 450 (Affymetrix) and then scanned with the GeneChip Scanner 3000 (Affymetrix). The entire experiment was performed twice. Array hybridization, washing, and scanning of the slides were carried out according to the manufacture’s protocols. The microarray numerical values were analyzed using the GeneSpring GX 11.0 software: quantile normalization, filter by flags (detected), filter by expression on the normalized data (20.0–100.0th percentile). Analysis of variance was used to determine those probe sets significantly different between the two groups. The gene list was filtered with a fold-change cutoff of 2, resulting in output of list with genes that have significant differential expression at 2-fold or more differences.

### Target Prediction of miRNAs

Basic Local Alignment Search Tool (BLAST), TargetScan 6.0, microRNA.org, was used to search for predicted the target genes of miRNAs.

### Prediction of binding site and mature miRNA transfection

Among the predicted target genes of miR-93 in the TargetScan (http://www.targetscan.org/), DIANA (http://diana.cslab.ece.ntua.gr/microT/), and PicTar (http://pictar.mdc-berlin.de/) databases, we found that PTEN was one of the top candidates. One day prior to the transfection, cells were seeded onto 6 well plates (1 × 10^5^ cells/well) and incubated with the complete medium without antibiotics (2 ml/well).　 Actinomycin D (10 μg/ml, Sigma-Aldrich) was used to inhibit nascent RNA synthesis. The transfection of miR-93-5p mimic (5′- CAAAGUGCUGUUCGUGCAGGUAG-3′) (20nM), miR-93-5p mutant (5′- CUUUCACGUGUUCGUGCAGGUAG-3′) (20nM) and negative control (NC) mRNAs (20nM) (Invitrogen) was performed using Lipofectamine 2000 reagent (Invitrogen) in antibiotics-free OptiMEM (Invitrogen) according to the manufacturer’s instructions. After 48 h incubation following the transfection, the cells were harvested and processed for further analysis.

### RNA extraction, cDNA synthesis, and quantitative real time PCR

Total RNA was extracted from treated cells with the TRizol reagent (Invitrogen) and cDNA was synthesized according to the manufacturer’s protocol (Roche). Quantitative real-time PCR (qRT-PCR) was performed using a Light Cycler 480 Probe Master System (Roche), and PCR-specific amplification was conducted in the LightCycler® Nano (Roche). The relative expression of genes (PTEN and GAPDH) was calculated with the 2-(ΔΔCt) method. The primers used are listed here (qRT-PCR; PTEN-forward 5′-AAGACAAAGCCAACCGATAC-3′, PTEN-reverse 5′-GAAGTTGAACTGCTAGCCTC-3′; GAPDH-forward 5′-CCTCTATGCCAACACAGTGC-3′, GAPDH-reverse 5′-GTACTCCTGCTTGCTGATCC-3′.

### miRNA inhibitor transfection

One day prior to the transfection, Saos and MG63 cells were seeded onto 6 well plates (1 × 10^5^ cells/well) and incubated with the complete medium without antibiotics (2 ml/well). The transfection of hsa-miR-93 inhibitor (5, 10, 20, 40, 80nM) or negative control miRNAs (Control-miR) (5, 10, 20, 40, 80nM) (Invitrogen) was performed using Lipofectamine 2000 reagent (Invitrogen) in antibiotics-free OptiMEM (Invitrogen) according to the manufacturer’s instructions. After 48 h incubation following the transfection, the cells were harvested and processed for further analysis.

### Cell proliferation assay

The PTEN expression plasmid (SC119965) was obtained from OriGene Tech. Inc (Iowa, USA). The Saos and MG63 cells were plated in 6-well plates (1 × 10^5^ cells per well), and were transfected with or without anti-miR-93 inhibitor, negative control miRNA, PTEN plasmid and Mock plasmid vector using Lipofectamine 2000. Then the cells were incubated in antibiotic-free OptiMEM. After 48 h of the cultivation, the cells were counted using TC10 Automated Cell Counter (BioRad).

### Western blot

Total cellular protein (15 μg) was resolved on a precast 10 % Tris–HCl Criterion 10-well gel (Biorad) at 200 V (300 mAmp) for 30 min. The gel was wet-transferred to a PVDF membrane for 1 h, and blocked with PBST containing 5 % instant dry non-fat milk for 30 min at room temperature. Primary antibodies (×1000) Rabbit source (1000) against PTEN (#9552), β-Actin (#4970), Akt (#4691), p-Akt (#4060), p21 (#2947), Bad (#9292) and p-Bad (#9291) proteins were obtained from Cell Signal Technology (Tokyo, Japan). Antibodies Rabbit source against p-p21 (ab47300) was obtained from Abcam (Tokyo, Japan). Immunocomplexes were visualized with horseradish peroxidase-conjugated anti-rabbit immunoglobulin G antibodies (×1000) (GE Healthcare, Tokyo, Japan), developed the blots using ECL Prime system (GE Healthcare) with a ChemiDoc camera (ImageQuant LAS 4000mini; GE Healthcare). The quantification of western blot signals was performed by the densitometry with ImageQuant TL software (GE Healthcare). All western blot experiments were repeated at least three times.

### Cell cycle analysis

For cell cycle analysis, Saos cells were stained with propidium iodide using Cycletest Plus DNA Reagent Kit (BD Biosciences) following the manufacturer’s protocol, and the cell cycle distribution was analyzed by FACSVerse flowcytometer (BD Biosciences). The percentages of cells in G0⁄G1, S, and G2⁄M phases were counted and compared. The experiments were carried out in triplicate.

### Apoptosis assay

The changes in the expression of apoptotic proteins were analyzed by western blot analysis using antibodies against PAR/poly (ADP-ribose) polymerase (PARP) (#9542) and cleaved PARP (#9541) (Cell signaling Tech, Tokyo, Japan) as an index of apoptosis.

The quantification of cell death was determined by fluorescence activated cell sorting (FACS) using Annexin V-FITC apoptosis detection kit (BD Bioscience) according to the manufacturer’s instructions. Briefly, 1 ×  10^6^ Saos cells were seeded and incubated for 24 h, then anti-miR-93 or PTEN expression plasmid was added to the cells followed by incubation for 48 h. The cells were washed with PBS, suspended in annexin V binding buffer, then added to an annexin V-FITC solution and propidium iodide (PI) for 20 min at room temperature. The samples were analyzed by FACSVerse using FACSuite analysis software (BD Bioscience). Adriamycin (ADM) is commonly used for the induction of apoptosis as positive control. To verify the ability for induction of apoptosis, Saos cells treated with low dose ADM at 5 μg/ml for 24 hours were used.

### In vivo tumor bearing nude mice models

The experimental tumor bearing model was established by injection of 2 x 10^6^ cells transfected with anti-miR-93 suspended in 100 μl of normal saline into the gluteal region of nude mice (BALB/c nu/nu, Kudo, Tosu, Japan). Four groups were generated: (1) untreated control (n = 7); (2) transfected with negative control-miRNA (n = 7); (3) transfected with anti-miR-93 (n = 7); and (4) transfected with PTEN expression vector (n = 7). All mice were fed in standard condition with weight monitoring and sacrificed 6 weeks after the cell inoculation. Tumor size was measured in two perpendicular dimensions parallel with the surface and the depth of the tumor in mice using a caliper. The primary tumor volume was estimated using the formula V = (Length × Width^2^)/2.

### Immunofluorescence microscopy

To determine the effect of anti-miR-93 and PTEN expression vector on the protein level of PTEN, we also performed immunofluorescence staining with PTEN (CST) or p-Akt antibodies (CST). After 48 hours, the transfected Saos cell lines were fixed with 4 % formaldehyde for 20 minutes, then incubated with 0.5 % Triton X-100. A rabbit anti-Human PTEN/p-Akt antibody was used for immunofluorescence staining. Following three washes with PBS, the cells were incubated with a goat anti-rabbit Alexa Fluor 594 antibody (Life Technology).

### Statistical analysis

Statistical analysis was carried out using SPSS 18.0 software (SPSS Japan Inc, Tokyo, Japan). Two-tailed student’s *t*-test was used for analysis of continuous variables. We determined the differences among more than 3 groups using a nonrepeated measures analysis of variance (ANOVA) and Scheffe test. Results were expressed as the mean ± standard deviation, and p < 0.01 was considered as statistically significant.

## Result

### Up regulation of miR-93 expression in OS cell lines

**Fig. 1:**
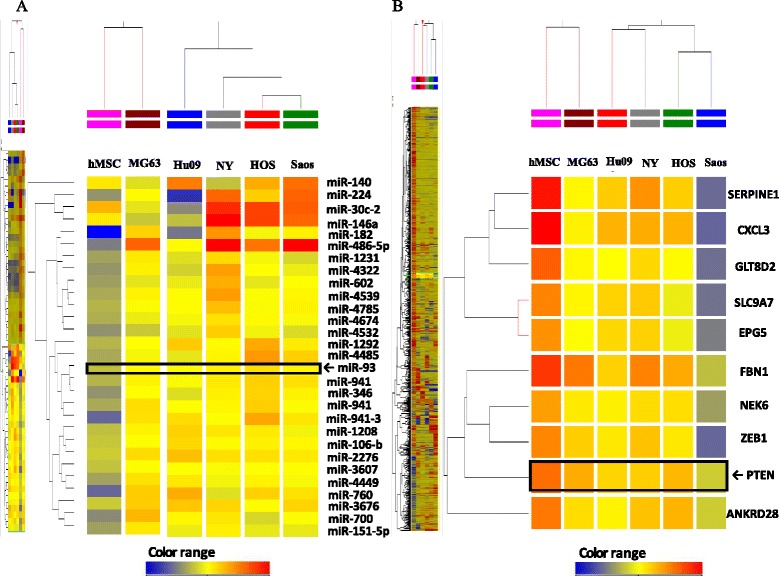
microRNA and cDNA array analysis in OS cell lines. **a** Heat map of genome-wide miRNA profile in five OS cell lines and hMSCs. miR-93 was down-regulated in all five OS cell lines. **b** Whole genome mRNA profile and extraction of PTEN and related genes as same pathways in OS cells and hMSCs. The color bar represented the grades of the relative expression levels; increase was represented by red while decrease was indicated by blue

The genome-wide miRNA expression profiling using five OS cell lines was carried out to identify miRNAs specifically expressed in OS cells. The array analysis showed that the expressions of 435 miRNAs in OS cells were changed (fold-change >2.0) in comparison with hMSCs (Fig. 1a). Among 435 miRNAs, 186 were significantly upregulated, whereas 170 were significantly down regulated in all tested OS cells compared with hMSCs. The remaining 79 miRNAs were upregulated or down regulated among the five OS cell lines. In OS cell lines, the increased expression of miR-93 by 2.22 ~ 3.57 folds compared with hMSCs was observed.

### Down regulation of PTEN expression in OS cell lines

The cDNA array analysis demonstrated that the expressions of 761 mRNAs were significantly changed between the five OS cell lines and hMSCs (Fig. 1b). We found that 124 genes were significantly upregulated, 448 genes were significantly down regulated, and the remaining 189 genes were up or down regulated in the five OS cell lines compared with hMSCs. The expression of PTEN was decreased by −2.05 ~ − 9.95 fold change in five OS cell lines.

### PTEN as a direct miR-93 target in OS cell lines

**Fig. 2:**
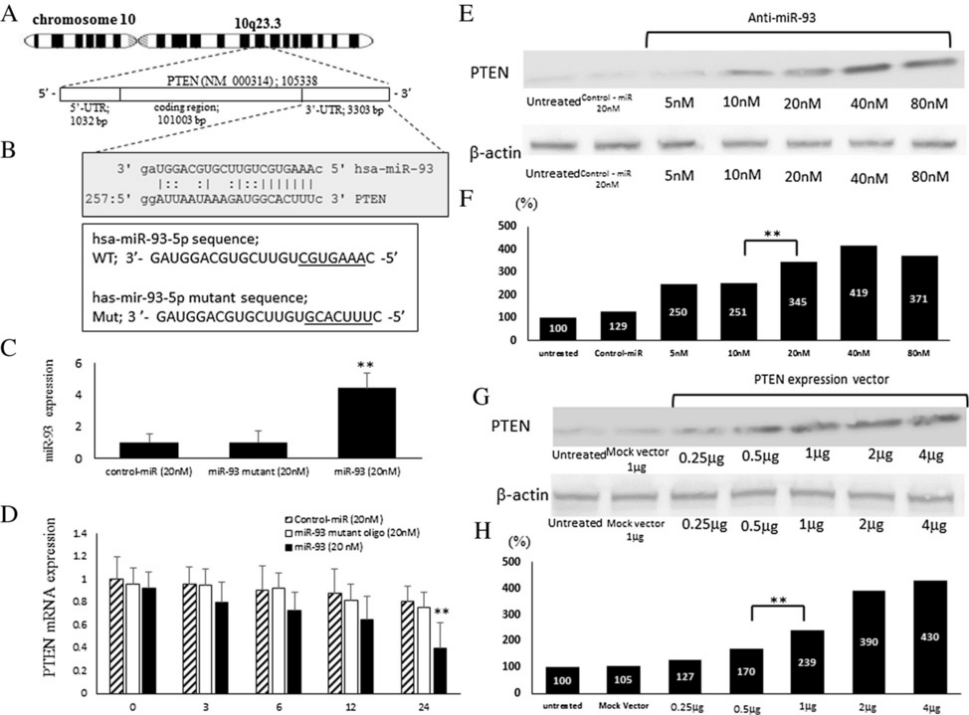
miR-93 inhibits PTEN mRNA expression in Saos cells and silencing of PTEN with anti-miR-93 in OS cells. **a** Predicted binding sites of miR-93 at the 3′UTR site of PTEN, as aligned by Target Scan and BLAST. **b** Scheme and sequence of the intact miR- 93 (Wt) and its mutant (Mut). (**c**) After actinomycinD treatment, the miRNA expression level of miR-93 in the negative control-miR, miR-93 and miR-93 mutant was measured by qRT-PCR. **d** After actinomycinD treatment, the mRNA expression level of PTEN after transfection of negative control-miR, miR-93 and miR-93 mutant was measured by qRT-PCR. **e** Up-regulation of PTEN using anti- miR-93 and PTEN-expression vector in ES cells. **f** Densitometry quantification of PTEN protein after transfection of anti-miR-93. **g** Transfection of PTEN expression vector in ES cells increases the expression of PTEN protein. **h** The quantification of PTEN protein after transfection of PTEN expression vector. ANOVA was carried out to statistically analyze the results. (**) P < 0.01

The region complementary to the miR-93 seed region was found in the 3′-UTR of human PTEN (Fig. 2a). A considerable complementarity between sequences within the seed regions of miR-93 and sequences in the 3′ untransrated region (UTR) of PTEN was predicted, using the algorithms in BLAST and TargetScan. The results suggested that miR-93 might affect the expression of PTEN genes by binding to 3′UTR of PTEN.　We blocked de novo mRNA transcription using actinomycin D (10 μg/ml), an inhibitor of mRNA transcription to determine changes in miRNA or mRNA stability. To test whether miR-93 expression affected endogenous PTEN expression, we transfected the miR-93 and miR-93 mutant oligonucleotides, as well as the negative control-miR, into Saos cells (Fig. 2b). We observed an increased miR-93 expression by 4.41 ± 0.96 fold compared with miR-93 mutant (0.99 ± 0.71) or control-miR (1.00 ± 0.53) (*p* < 0.01) (Fig. 2c). And decreased PTEN expression at the mRNA level following transfection with the miR-93-5p oligonucleotide 0.4 ± 0.22 compared with miR-93 mutant (0.75 ± 0.14) or control-miR (0.81 ± 0.13) (*p* < 0.01) (Fig. 2d).

### Up-regulation of PTEN expression by anti-93 miRNA and PTEN expression vector

To examine the correlation between miR-93 and PTEN in OS cells, miR-93 inhibitor anti-miR-93 was transfected into Saos cells. Western blot analysis showed that the expression levels of PTEN dramatically increased in anti-miR-93 transfected cells compared with untreated or negative control-miR transfected cells (Fig. 2e). The protein expression level of PTEN in the anti-miR-93 (20nM) transfected cells was increased to 3.45 folds of that in the control cells (*p* < 0.01) (Fig. 2f). To further confirm the effects of PTEN on the growth of OS cells, the transfection with PTEN expression vector was carried out. Although the expression level of PTEN protein in the cells transfected with Mock vector was not significantly affected, that in the cells transfected with PTEN expression vector was significantly reduced, as determined by Western blot (Fig. 2g). Compared to the control cells (100 %), PTEN vector (1 μg) transfected cells exhibited the significantly higher PTEN expression level by 2.39 folds (*p* < 0.01) (Fig. 2h).

### Suppression of OS cell growth by transfection of anti-93-miR and PTEN plasmid vector

**Fig. 3:**
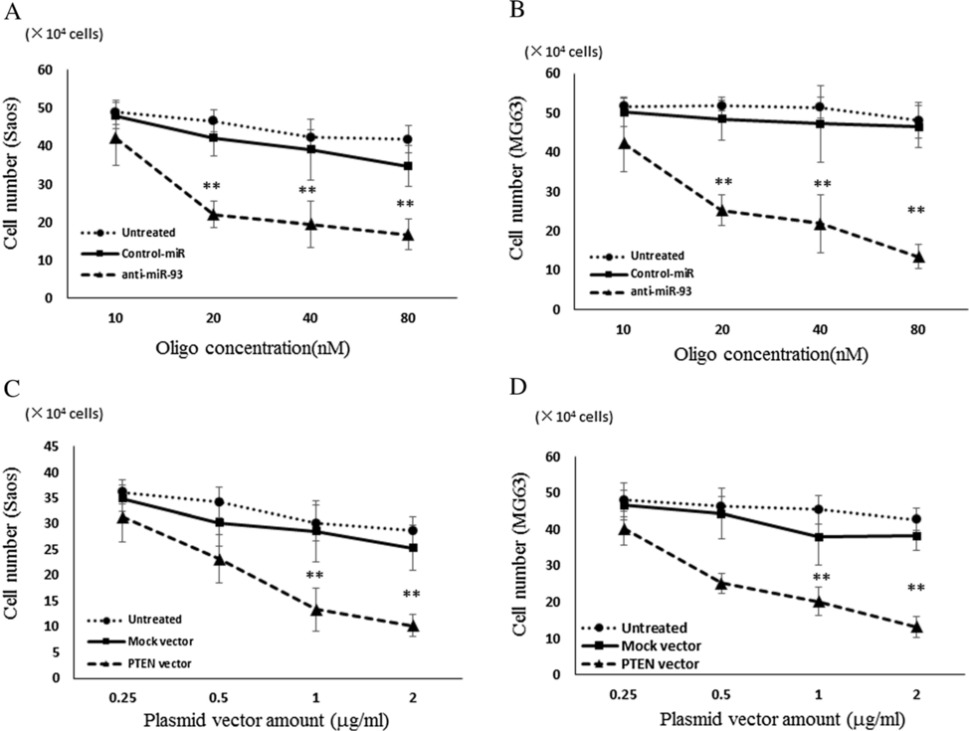
Cell proliferation assay performed to evaluate the anti-proliferation effect. Cell proliferation assay to evaluate the antiproliferation effect of anti-miR-93 (**a**) and (**b**) and PTEN vector (**c** and **d**) in OS cells. Error bars represent mean ± S.D. from three independent experiments. The two-tail student *t*-test was employed to statistically analyze the results: (**) P < 0.01

PTEN is known to play important roles in the regulation of cell proliferation. Since the transfection of anti-miR-93 resulted in the reduction of PTEN expression, we next examine the effects of anti-miR-93 on the proliferation of ES cells. The cell growth of Saos (Fig. 3a) and MG63 (Fig. 3b) was inhibited by the transfection of anti-miR-93 as determined by cell counting in comparison with untreated and negative control-miRNA transfected cells at 48 h after the transfection. PTEN plasmid vector transfected Saos (Fig. 3c) and MG63 (Fig. 3d) cells, as same as anti-miR-93 transfected cells, showed significant inhibition of the cell proliferation compared with the untreated and mock plasmid vector transfected cells.

### PTEN restoration induced the expression change of Akt

**Fig. 4:**
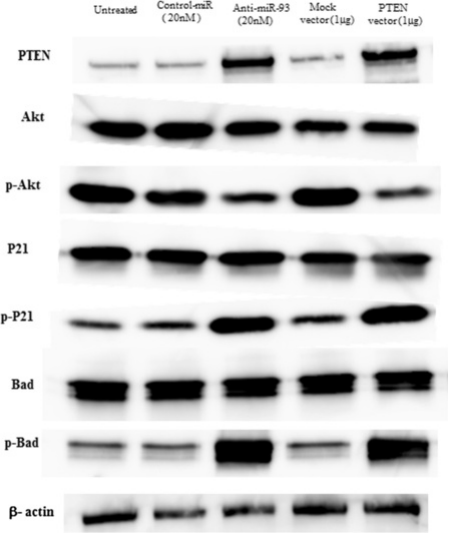
A western blot analysis of PTEN, Akt and other factors related to cell cycle and apoptosis. Effect of anti-miR-93 and PTEN expression vector on PTEN protein expression and its downstream factors in Saos cells after 48 hours of transfection

To examine the correlation between PTEN and Akt, the expression of PTEN, Akt, and their downstream factors were investigated in Saos cells after 48 hours of transfection. Saos transfected miR-93 inhibitor and PTEN vector showed the increase in expression of PTEN, phospho (p)-p21 and p-Bad. Western blot analysis showed that the expression levels of p-Akt dramatically decreased in anti-miR-93 and PTEN vector transfected cells compared with untreated, negative control oligo and Mock vector transfected cells (Fig. 4).

### Induction of cell cycle arrest at G0/G1 phase by anti-miR-93

**Fig. 5:**
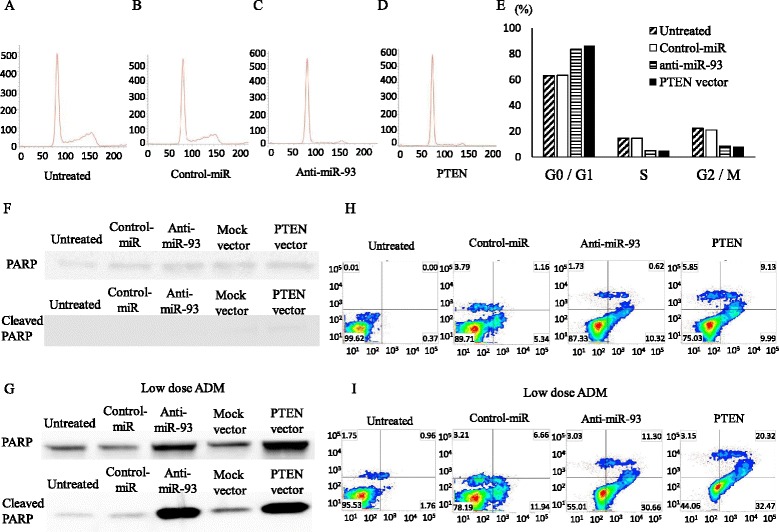
Effects of anti-miR-93 and PTEN expression vectors on the cell cycle distribution in Saos. Cells were treated and analyzed by flow cytometry after staining with PI (**a-d**). Histogram shows quantitative percentage of diploid cells (DNA content) in each cell cycle phase (**e**). Effects of anti-93-miR and PTEN vector on induction of apoptosis in Saos. Western blot showed the expression of PARP and its cleaved form (**f**). Transfection of anti-93-miR and PTEN plasmid with low dose Adriamycin showed the expression of PARP and its cleaved form Saos (**g**). The cells were labelled with FITC annexin V and PI in oligo transfection (**h**) and oligo transfection with low dose Adriamycin (**i**). Each quadrants represent viable cells (Lower Left quadrant), early apoptotic cells (Lower Right), late or secondary necrotic cells (Upper Right), and primary necrotic cells (Upper Left), respectively

Since the introduction of anti-miR-93 significantly inhibited cell proliferation of OS Saos cell lines, we hypothesized that anti-miR-93 might reduce the cell cycle progression and⁄or induce-apoptosis of the cells. To monitor the cell cycle distributions, FACS analyses were carried out using anti-miR-93 and PTEN plasmid transfected cells (Fig. 5a-d). Both in anti-93-miR and PTEN plasmid transfected cell lines, the number of the cells in G2⁄M and G0/G1 phase was significantly lower and higher than that in the control cells, respectively (Fig. 5e). The data suggested that the repression of miR-93 and restoration of PTEN might have resulted in G0⁄G1 retardation in OS cells. Then the cellular expression of PARP and its cleaved product was assayed by immunoblotting in Saos cells and their transfectants (Fig. 5f). The cleavage of PARP protein, a marker of caspase‑mediated apoptosis, was not observed in both anti-miR-93 and PTEN transfectants as well as untreated cells and negative control or mock plasmid vector transfectants. Additional low dose ADM treatment could induced expression of cleaved PARP in both anti-93-miR and PTEN transfectant, which were not observed in untreated cells, negative control and mock plasmid vector transfectants. (Fig. 5g). Furthermore, in flow cytometry analysis using Annexin V-FITC/PI double staining, there were no significantly differences in the distribution patterns between untreated, negative control miRNA, anti-miR-93 and PTEN plasmid transfected cells (Fig. 5h). The programmed cell death was induced by low dose ADM treatment in anti-miR-93 or PTEN plasmid vector in Saos cells (Fig. 5i).

### Inhibition of tumor growth in nude mice xenograft model by anti-93-miR

**Fig. 6:**
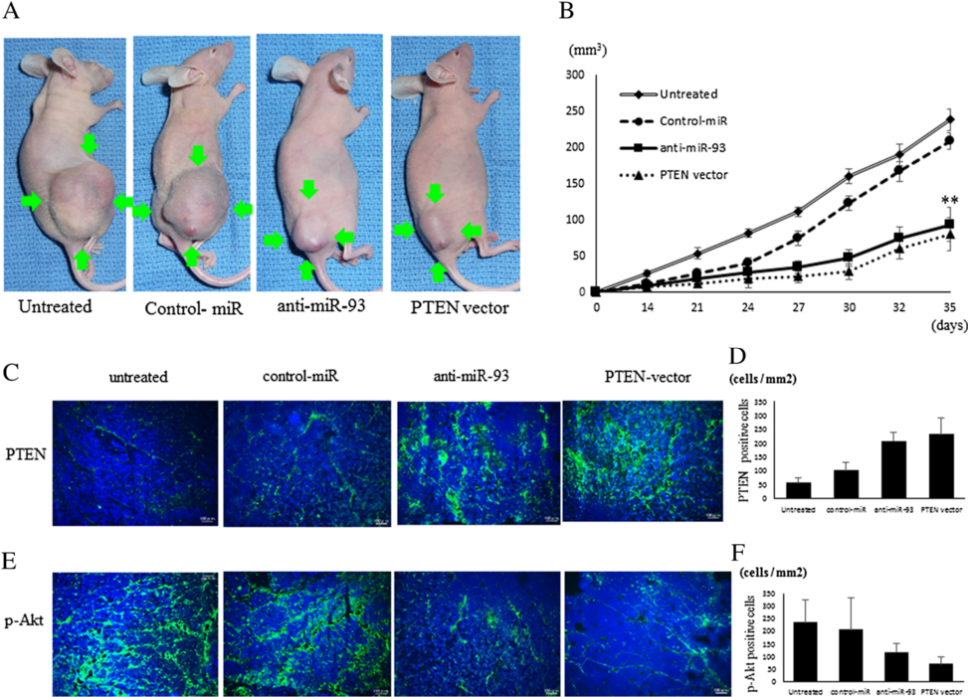
Anti-miR-93 and PTEN vector suppressed in vivo tumor growth. Four groups were; (1) untreated (n = 7); (2) transfected with of negative control-miRs (n = 7); (3) transfected with anti-miR-93 (n = 7); and (4) transfected with PTEN expression vector (n = 7). Tumor volumes were measured at the time points indicated after the tumor cell inoculation. (**) P < 0.01. Immunofluorescence of PTEN (**c**) and p-Akt (**f**) following transfection of anti-miR-93 and PTEN vector into tumor cells. The number of PTEN (**d**) and p-Akt (**f**) positive cells per unit area in the tumor lesion

We next investigated the efficacy of anti-miR-93 against tumor growth in vivo. The introduction of anti-miR-93 into Saos cells resulted in the decreased growth of subcutaneous xenografted tumors in nude mice (Fig. 6a). Saos cells transfected with anti-miR-93 showed statistically smaller tumors in mice compared to untreated and negative control miRNA transfected groups (Fig. 6b.), indicating that anti-miR-93 also inhibited the growth of OS cells in vivo.

The expression of PTEN (Fig. 6c) and p-AKT (Fig. 6e) within the primary tumor lesion was reduced in the anti-miR-93 and PTEN expression vector transfected tumor tissues. The number of PTEN positive cells per unit area was higher (p < 0.01) in the mice that anti-miR-93 (208.3 ± 30.8 cells/mm2) and PTEN expression vector 233.8 ± 57.9 cells/mm2) than in those that received untreated (56.7 ± 16.7 cells/mm2) and control-miR (101.5 ± 28.1 cells/mm2) (Fig. 6d). The number of p-Akt positive cells per unit area was lower (p < 0.01) in the mice that ati-miR-93 (117.6 ± 31.1 cells/mm2) and PTEN expression vector (68.8 ± 25.1 cells/mm2) than in those that received untreated (234 ± 42.2 cells/mm2) and control-miR (206.2 ± 57 cells/mm2). (Fig. 6f).

## Discussion

miRNAs are small (19–25 nucleotides), noncoding RNAs capable of modulating the expression of their cognate target genes by binding to the 3′-UTR of mRNAs, causing either translational inhibition or mRNA cleavage [3, 4]. They are expressed in a tissue-specific manner and play an important role in cell proliferation, apoptosis, and differentiation. It has been shown that miRNAs play crucial roles in the regulation of diverse biological processes, such as development, inflammation, and tumorigenesis [14]. miRNAs are frequently dysregulated in human cancers and can act either as potent oncogenes or as tumor suppressors [15, 16]. To identify important miRNA–mRNA relationships in OS, we performed genome-wide miRNA and cDNA arrays on the same OS cells.

In the present study, our miRNA array results demonstrated that the expression of miR-93 was up-regulated in all five OS cell lines. miR-93 overexpression plays an important role in promoting lung cancer cell growth, angiogenesis, and metastasis, while its inhibition suppresses cell proliferation, migration, and colony formation [17, 18]. Furthermore, additional in vitro and in vivo experiments have demonstrated the critical role of miR-93 in regulating cancer cell growth and promotion of tumor progression [19, 20]. The function of miR-93 has been reported to be important in the ecology of other types of cancer via targeting of PTEN [5, 20, 21]. However, the biological role of miR-93 including the relationship to PTEN in OS cells has not yet been clarified. As our results indicated that miR-93 expression was consistently up-regulated in OS cell lines, we employed genome-wide mRNA profiling by cDNA array to identify possible targets of miR-93 in OS cells.

Data from cDNA arrays showed that PTEN mRNA expression was invariably diminished in OS cell lines, in comparison to hMSCs. Furthermore, sequence analysis suggested a possible association of miR-93 with the 3′-UTR of PTEN. PTEN is a plasma membrane lipid phosphatase and tumor suppressor that dephosphorylates phosphatidylinositol 3,4,5-trisphosphate to the biphosphate phosphatidylinositol 4,5-bisphosphate, thereby inhibiting the phosphorylation of Akt [22, 23]. Studies of the Akt pathway have shown that a chromosome 10 deletion of phosphoinositide 3-kinase (PI3K)/PTEN can facilitate tumorigenesis [24, 25]. Our data, generated using OS cells, is consistent with that of previous studies showing that down-regulation of PTEN may contribute to malignancy.

Although miR-93 may influence the expression of many genes, we focused on PTEN as its target in OS cells. Several genes have been reported to be targets of miR-93, including *RhoC, CCNG2, RBL2, NRF2, and DAB2* [17, 19, 26–28]. Our cDNA array analysis demonstrated that PTEN was the only miR-93 target gene whose expression was uniformly up-regulated in all five OS cell lines. The expression of the other candidate genes differed between these lines. An analysis using several algorithms, such as BLAST and TargetScan, further recommended PTEN as a putative target of miR-93. Therefore, we considered the possibility that miR-93 might affect anticarcinogenic mechanisms by targeting PTEN in OS cells. It is possible that miR-93 may down-regulate PTEN expression via an indirect pathway. In order to examine whether miR-93 really inhibits PTEN mRNA function, we stopped new transcription with actinomycin D and then introduced miR-93 and a mutant with a changed seed region. The miR-93 mutant did not result in any changes, whereas the miR-93 introduction group demonstrated a reduction in PTEN mRNA; therefore, miR-93 sequence can indeed be anticipated to directly inhibit PTEN mRNA function. Therefore, miR-93 may have affected PTEN mRNA through direct interaction, at least in part.

We next examined the function of miR-93 in regulating its potential target gene, PTEN, and in modifying the biological characteristics of OS cells. The over-expression of miR-93 resulted in the suppression of PTEN protein levels, indicating that miR-93 might function as an oncogene in OS cells. Several studies have demonstrated that PTEN, a cell cycle and proliferation regulator, is the direct target of miR-93 in breast cancer [5, 20, 29]. Our results suggest for the first time that the same regulatory mechanism may operate in OS cells. The expression of miR-93 has been identified in various types of cancer, suggesting an oncogenic role [6, 30, 31]. For instance, its overexpression in breast cancer has been correlated with proliferation and tumor progression [8].

Our data show that miR-93 promoted the proliferation of OS cells via induction of cell cycle retardation at the G1/G0 phase. This observation is consistent with a previous report demonstrating that overexpression of miR-93 promotes glioma cell proliferation and cell cycle progression [30, 31]. It has been shown that PTEN plays important roles in regulating G1/S transition and cellular transformation, and that restoring PTEN expression results in G1-phase cell cycle arrest [32]. Thus, we can assume that up-regulation of miR-93 might affect OS cell cycle progression via control of PTEN expression. It is noteworthy that up-regulation of PTEN by transfection with vectors encoding anti-miR-93 or PTEN itself induced apoptosis of OS cells, indicating that the repression of OS cell growth following restoration of PTEN expression came about by apoptosis, as well as by cell cycle retardation.

Given that PTEN acts as a major negative regulator of the PI3K/Akt signaling pathway, its inhibition by miR-93 might play an important role in the proliferation of other cell types [20]. Akt represses p21 and Bax, promoting cell proliferation and preventing apoptosis [33, 34]. Thus, down-regulation of Akt by PTEN in ES cells may increase p21 and Bax expression, leading to cell cycle retardation and apoptosis of OS cells.

Furthermore, the repression of miR-93 resulted in inhibition of OS tumor growth in vivo. Consistent with data from our in vitro experiments, the xenograft model of OS also indicated that this mechanism operates via restoration of PTEN expression.

## Conclusion

In summary, the present study demonstrates for the first time an inverse correlation between miR-93 and PTEN in OS cells. In addition, we have provided evidence that the expression of miR-93 in OS cells is significantly increased, and that this miRNA plays important roles in OS cell proliferation and tumorigenesis by targeting PTEN both in vitro and in vivo. The silencing of PTEN has been observed in several human cancers, in which it potently promotes cell growth and proliferation [35]. Our data suggest that PTEN expression constitutes one of the crucial factors determining tumor proliferation in ES, as in other malignant tumors. Although future confirmation of the data presented in the current study using clinical OS samples is necessary, the novel information provided here regarding the link between miR-93 and PTEN in OS cells will be beneficial in better understanding ES oncogenesis, and may suggest certain therapeutic strategies for clinical application.
